# Influence of Whole-Body Electrostimulation on the Deformability of Density-Separated Red Blood Cells in Soccer Players

**DOI:** 10.3389/fphys.2019.00548

**Published:** 2019-05-09

**Authors:** Andre Filipovic, Daniel Bizjak, Fabian Tomschi, Wilhelm Bloch, Marijke Grau

**Affiliations:** Institute of Molecular and Cellular Sports Medicine, German Sport University Cologne, Cologne, Germany

**Keywords:** electrostimulation, soccer, RBC, VO_2_peak, NO-oxidation, deformability

## Abstract

Red blood cell nitric oxide synthase (RBC-NOS) dependent NO production positively affects RBC deformability which is known to improve oxygen supply to the working tissue. Whole-body electrostimulation (WB-EMS) has been shown to improve maximum strength, sprinting and jumping performance, and to increase deformability in elite soccer players during the season. The aim of the present study was to investigate whether WB-EMS affects RBC turnover which might affect overall deformability of circulating RBC by rejuvenation of the RBC population and if this might be related to improved endurance capacity. Thirty male field soccer players were assigned in either a WB-EMS group (EG, *n* = 10), a training group (TG, *n* = 10), or a control group (CG, *n* = 10). EG performed 3 × 10 squat jumps superimposed with WB-EMS twice per week in concurrent to 2–4 soccer training sessions and one match per week. TG only performed 3 × 10 squat jumps without EMS in addition to their soccer routine and the CG only performed the usual soccer training and match per week. Subjects were tested before (Baseline) and in week 7 (wk-7), with blood sampling before (Pre), 15–30 min after (Post), and 24 h after (24 h post) the training. Endurance capacity was determined before and directly after the training period. The key findings of the investigation indicate an increase in young RBC in the EG group along with improved overall RBC deformability, represented by decreased SS1/2:EImax Ratio. Analysis of the different RBC subfractions revealed improved RBC deformability of old RBC during study period. This improvement was not only observed in the EG but also in TG and CG. Changes in RBC deformability were not associated to altered RBC-NOS/NO signaling pathway. Endurance capacity remained unchanged during study period. In summary, the effect of WB-EMS on RBC physiology seems to be rather low and results are only in part comparable to previous findings. According to the lower training volume of the present study it can be speculated that the soccer specific training load in addition to the WB-EMS was too low to induce changes in RBC physiology.

## Introduction

Electromyostimulation (EMS) has been used to complement rehabilitation programs for many years. Lately, EMS is increasingly combined with strength training in high performance sports. Modern whole-body EMS (WB-EMS) systems (e.g., miha bodytec, Augsburg, and Germany) allow athletes to simultaneously stimulate several muscle groups, to train a whole muscle chain and thus to dynamically train specific movements, e.g., jumping movement. A recent study with professional soccer players revealed an increase in maximal strength, jumping, and sprinting ability after WB-EMS training (Filipovic et al., 2016). The study further showed that two sessions of dynamic WB-EMS a week can be sufficient to significantly influence the functional parameters of the red blood cells (RBC) (Filipovic et al., 2015).

Within the body, RBC deliver oxygen to the muscle tissues via the blood flow through the vessel system. To do so, RBC have to deform their shape in order to pass the smallest capillaries of the microcirculation. This RBC deformability is a unique cell characteristic and is, among others, influenced by nitric oxide (NO) (Bor-Kucukatay et al., 2003; Suhr et al., 2012; Grau et al., 2013). In RBC, NO is actively produced by RBC-NO synthase (RBC-NOS) (Kleinbongard et al., 2006; Grau et al., 2013). The phosphorylation status of RBC-NOS has been used as a marker of enzyme activation. Activation occurs through different stimuli such as inflammatory cytokines, growth factors, and hormones, etc (Forstermann and Sessa, 2012) or exercise induced shear stress through activation of Akt kinase (Suhr et al., 2012). Biomechanical stimulation in the form of increased shear stress stimulates the phosphorylation of the RBC-NOS epitopes serine 1177 (Serine^1177^) via the PI3 Kinase/Akt Kinase pathway (Dimmeler et al., 1999; Suhr et al., 2012). The activated RBC-NOS generates NO, which is a precondition for increasing RBC deformability (Suhr et al., 2012; Grau et al., 2013). In contrast, phosphorylation of RBC-NOS residues threonine 495 (Thr^495^) or serine 114 (Ser^114^) were associated to decreased RBC-NOS activation (Grau et al., 2016). RBC-NOS produced NO binds to reactive cysteine thiols, a reaction termed S-nitrosylation. Grau et al. (2013) identified α- and β-spectrin as potential targets for S-nitrosylation in the RBC with increasing S-nitrosylation of the spectrins being associated to increased RBC deformability.

Connes et al. (2004) indicated that increased RBC deformability might improve the blood oxygen content due to an increased oxygen diffusion from alveoli to pulmonary capillaries. This might suggest that an increase in RBC deformability might favour performance capacity.

Soccer match play is characterized by high intensity repeated sprint actions that require a high muscle oxygenation. Higher muscle oxygen-level can positively influence the re-oxygenation of the muscles and thus phosphocreatine re-synthesis for a faster recovery. Due to the high demand of muscle oxygenation during match play, improved RBC deformability could be advantageous for the specific endurance capacity of soccer players such as repeated sprint ability (c.f. Brun et al., 2010).

An increase in RBC deformability after WB-EMS stimulation was associated – at least after acute application – via RBC-NOS activation and increased NO production, respectively (Filipovic et al., 2015). However, the observed chronic increase in RBC deformability occurred in the absence of a further RBC-NOS activation and it was speculated whether WB-EMS might affect RBC turnover. RBC are a heterogeneous cell population consisting of RBC of different ages. RBC aging was associated to a progressive decrease in RBC deformability, paralleled by increasing RBC-NOS activation and NO production (Bizjak et al., 2015).

The purpose of the present study thus was to investigate whether a 7 week dynamic WB-EMS program affects RBC deformability through shift in RBC age distribution, and to examine whether these changes are sustained 3 weeks after the last intervention session. Further, it is unknown if an increased RBC deformability through WB-EMS-Training can positively influence the endurance capacity which was thus also aim of the present study.

## Materials and Methods

### Participants

Only healthy participants were included which means no cardiovascular or metabolic diseases and no preinjury in the tested muscle groups. Participants needed to compete on a national level for the last 3 years and train 2–4 session per week and play one soccer match per week. Experience in strength training was mandatory. Thirty soccer players were randomly assigned into three different groups. The EMS groups (EG, *n* = 10) performed dynamic whole-body strength training with EMS twice a week accompanied by 3 × 10 squat jumps in addition to the daily soccer routine over a period of 7 weeks. To differentiate between the effects caused by EMS and by the squat jumps and soccer training, respectively, two control groups were included. A jump training group (TG, *n* = 10) performed the same number of squat jumps without EMS stimulus on the same days as the EG and a control group (CG, *n* = 10) that only performed the daily soccer routine.

Basal anthropometric parameters of the participants are presented in Table 1. All subjects abstained from alcohol consumption for 24 h prior to and during the training intervention and were non-smokers.

**Table 1:** Anthropometric data (mean ± SD) and total training load (arbitrary units) during the 7-week intervention period calculated by Polar Team-2 Software according to training time spent in defined heart rates (see section “Materials and Methods”).

Group	Age (Year)	Height (m)	Weight (kg)	Bodyfat (%)	relVO_2_peak (ml/kg^∗^min^-1^)	Sessions/week	Total training load (a.u)
**EG**	24.4 ± 4.2	1.82 ± 0.03	81.4 ± 5.3	12.9 ± 2.1	52.1 ± 3.4	3.4 ± 1.2	3430.6 ± 910.7
**TG**	21.1 ± 1.9	1.83 ± 0.06	79.7 ± 5.5	10.8 ± 2.8	56.3 ± 5.7	3.4 ± 1.3	3478.6 ± 1722.8
**CG**	23.6 ± 3.9	1.82 ± 0.05	79.7 ± 7.5	14.1 ± 3.6	54.3 ± 7.2	2.6 ± 0.7	2644.4 ± 1437.3


This study was carried out in accordance with the recommendations of the Ethics Committee of the German Sports University Cologne. All subjects gave written informed consent in accordance with the Declaration of Helsinki. The protocol was approved by the Ethics Committee of the German Sports University Cologne [06-02-2014].

### Definition of Daily Soccer Routine

The participants were soccer players and performed 3.2 ± 1.0 training sessions per week and competed once a week in the championships. The standard training sessions lasted approximately 90 min including technical skill activities, offensive and defensive tactics, athletic components with various intensities, small-sided game plays, and continuous play. In a normal training week during season with a match on Sunday training was scheduled on Tuesdays, Wednesdays (optional), Thursdays, and Fridays. Number of training sessions and the training days varied according to the game schedule playing Sunday-Sunday or Sunday-Saturday. The number of training sessions and the total training minutes were documented. The training load was measured according to the training time spent in defined heart rate zones during soccer training or match via Polar Team-2 Software (Polar Electro, Büttelborn, Germany) (see Table 1). The training load provided by the Polar-Software aims to determine internal training load based on background variables [sex, training history, metabolic thresholds, and maximal oxygen consumption (VO_2_max)] and parameters measured during training sessions (exercise mode, and energy expenditure) (c.f. Schumann et al., 2017). The heart rate zones (100–90%, 89–80%, 79–70%, 69–60%, and 59–50%) were defined according to the individual maximum heart rate measured in the maximal ramp test (see endurance test).

The players were asked to maintain their usual food intake und hydration according to the recommendations for soccer players (García-Rovés et al., 2014) and no nutrition supplementation was used. Additional strength training was not allowed during the study.

All players had a constant training volume during the first half of the season (July till December) and were in a well-trained condition with a relative VO_2_peak of 54.2 ± 5.9 ml/kg^∗^min^-1^. All players regularly conducted strength training during first half of the season and had experience in strength training of 5.4 ± 3.9 years. The intervention period started after the 3 week mid-season break from end of December till mid of January. During these 3 weeks the training load was relatively low (moderate endurance training twice per week) in order to maintain fitness level and not negatively affect baseline testing.

### Exercise Protocol

Whole-body electrostimulation training was conducted on Tuesdays and Fridays in order to obtain a rest interval of 48 h between the two sessions and the championship game on Sunday. The WB-EMS training was conducted using a WB-EMS-system by “*miha bodytec*” (Augsburg, Germany). WB-EMS was applied with an electrode vest to the upper body including the chest, upper and lower back, latissimus, and the abdominals and with a belt system to the lower body including the muscles of the glutes, thighs, and calves. Biphasic rectangular wave pulsed currents (80 Hz) were used with an impulse width of 350 μs (c.f. Filipovic et al., 2016). The stimulation design has been shown to be effective for enhancing strength and performance parameters and to also positively influence RBC deformability (Filipovic et al., 2015, 2016). The squat jumps were only included to integrate specific movement patterns to support strength transfer into jumping and sprinting ability (Requena-Sanchez et al., 2005) and for a better regulation of stimulation intensity to a sub-maximal level. The stimulation intensity (0–120 mA) was determined and set separately for each muscle group by using a Borg Rating of Perceived Exertion (c.f. Tiggemann et al., 2010). The training intensity was defined for each players in a familiarization session 2 weeks before and set at a sub-maximal level that still assures a clean dynamic jump movement (RPE 16–19 “hard to very hard”) and was saved on a personalized chip card. The EG performed 3 × 10 maximal squat jumps with a set pause of 60 s (no currents) per session. Every impulse for a single jump lasted for 4 s (range of motion: 2 s eccentric from standing position to an knee angle of 90° – 1 s isometric – 0.1 s explosive concentric – 1 s landing and stabilization) followed by a rest period of 10 s (duty cycle approx. 28%). The players started with a 2–3 min standardized warm-up with movement preparations including squats, skipping and jumps in different variations (squat jumps, jumps out of skipping, or double jumps) at a light to moderate stimulation intensity. The players were told to slowly increase the intensity every few impulses. The training started when the players reached the defined training intensity that was saved on the chip card from the last session according to the RPE 16–19 (“hard to very hard”). The stimulation intensity was constantly increased individually every week (Tuesdays) controlled by the coaches in order to maintain a high stimulation intensity. The intensity was increased after the warm-up during the first and the second set of 10 squat jumps starting from calves up to the chest electrodes.

The TG conducted the same standardized warm-up and performed the same amount of jumps with identical interval and conduction twice per week without EMS. The CG only performed the 2–4 soccer training session plus one match per week.

### Experimental Protocol

#### Endurance Test

**FIGURE 1:**
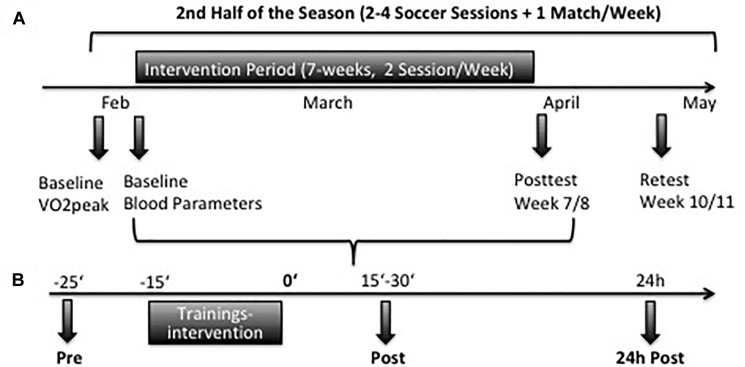
**(A)** Timeline of endurance testing and blood samples during the study in the second half of the season. **(B)** Timeline of blood samples collection at Baseline and in week 7. At each testing samples were taken before (pre), after 15–30 min (post), and 24 h (24 h post) after the training interventions.

For determination of relative maximum oxygen uptake (VO_2_ peak), spirometry was performed on a WOODWAY treadmill (Woodway GmbH, Weil am Rhein, Germany) 1 week before (Baseline) and within 7 days after the 7-weeks intervention period (Posttest) and again after a detrain period of 3 weeks (Retest) (Figure 1). Endurance tests were conducted three days after the soccer match to assure adequate recovery and not negatively influence performance. Spirometry was analyzed with the ZAN600-System and ZAN-Software GPI 3.xx (ZAN Austria e.U., Steyr-Dietach, Austria). To calibrate the device, a gas mixture consisting of 5% CO_2_, 16% O_2_, and rest nitrogen was used (*Praxair Deutschland GmbH*, Düsseldorf, Germany). To measure VO_2_ peak, the subjects performed an endurance ramp test according to a protocol of the Institute of Cardiovascular Research and Sports Medicine (German Sport University Cologne).

The players performed a warmup with moderate speed (3 m/s) at 1% incline for 3 min. In the last 30 s the incline was increased to 2.5%. Running speed was then increased every 30 s by 0.3 m/s till subjective exhaustion. Heart rate was documented in the last 10 s of a ramp stage. The VO_2_peak was determined as average maximum oxygen uptake of the first 20 s after completion of the test. Additionally, maximum heart rate (HF_max_), time to exertion (TTE), maximum lactate production and respiratory quotient (RQ = V CO_2_/V O_2_) was documented by the ZAN-system.

#### Blood Sampling

At Baseline and at wk-7, blood samples were taken from the vena mediana cubiti of EG and TG before (Pre), 15–30 min after (Post) and 24 h after the interventions (24 h Post), respectively. Blood samples of the CG were taken at the same day time as the intervention groups. For CG samples were only taken before (Pre) because no training sessions were performed between pre and 24 h post sampling (Figure 1). A third sampling was scheduled 3 weeks after the end of the intervention period (Retest). Blood was anticoagulated using ethylenediaminetetraacetic acid (EDTA) vacutainer (BD, United States) to measure basal blood parameters or using sodium heparin vacutainer (BD, United States) to measure RBC deformability of RBC in general and of RBC separated according to RBC age (young, main fraction, old, and very old) and related RBC-NOS/NO parameters such as S-nitrosylation and nitrite.

#### Basal Blood Parameters

Ethylenediaminetetraacetic acid anticoagulated blood was used for the determination of reticulocytes and basal blood parameters (RBC count, white blood cell count, platelet count, hemoglobin concentration, hematocrit, mean cellular volume, mean cellular hemoglobin, and mean cellular hemoglobin concentration). Reticulocytes were analyzed by laboratory Dr. Wisplinghoff, Cologne, Germany. Determination of the hematological profile was obtained using hematology analyzer Sysmex Digitana KX-21N (Sysmex, Horgen, Switzerland).

#### RBC Subpopulation

Red blood cell were isolated and fractioned to separate young, main fraction old and very old RBC using percoll density centrifugation according to a modified protocol of Bizjak et al. (2015).

Whole blood samples were centrifuged for 1 min at 3500 g at room temperature and plasma and buffy coat were removed. Isolated RBC were washed 10:1 with GASP-buffer (9 mmol/L Na_2_HPO_4_,_1.3_ mmol/L NaH_2_PO_4_, 140 mmol/L NaCl, 5.5 mmol/L glucose, 0.8 g/L BSA) and centrifuged as described above. The supernatant was discarded and RBC were diluted 1:1 with SAH-buffer (26.3 g/L BSA, 132 mmol/L NaCl, 4.6 mmol/L KCl, 10 mmol/L HEPES).

Percoll solutions with a density of 1.064, 1.066, 1.068, 1.072, and 1.076 g/mL in SAH buffer (Amersham Biosciences, Sweden) were prepared from a Percoll stock solution (1.131 g/mL; VWR). Solutions were layered into a 15 ml tube one on top of the other with the densest layer being right at the bottom. 600 μl of washed and diluted RBC were cautiously given on top of the layers centrifuged at 3000 rpm for 25 min at room temperature to receive young (1.064 g/ml), main fraction (1.065–1.068 g/ml), old (1.072 g/ml), and very old (1.076+ g/ml) RBC. RBC fractions were washed 1:1 with GASP and centrifuged as described above. The supernatant was discarded and the RBC fractions were used for deformability and age distribution analysis. The proportion of each fraction was determined and expressed as percentage of whole RBC.

#### RBC Deformability

Red blood cell deformability was directly measured after blood sampling and after separation of RBC according to cell age by ektacytometry using the Laser-assisted-optical-rotational cell analyzer (LORCA; RR Mechatronics, Netherlands) described in detail by Hardeman et al. (2001). Briefly, 10 μl of RBC were solved in 2.5 ml of isotonic 0.14 mM polyvinylpyrrolidone (PVP) (osmolarity 300 mOsmol/L, viscosity 28.7 mPa^∗^sec at 37°C). The samples were sheared in a Couette system at nine shear stresses between 0.3 and 50 Pa. A laser beam was directed through the samples and deformation of RBC affected diffraction pattern of the laser beam. The LORCA software used and width (W) and length (L) of the diffraction pattern to calculate an elongation index (EI): EI = (L-W)/(L+W) for the nine shear stresses. EI_max_, representing maximal deformability at infinite shear stress, and SS ½, representing shear stress necessary for one-half of EImax, were calculated from the curves according to Baskurt et al. (2009). Finally, the ratio of SS1/2:EImax was calculated as described by Baskurt and Meiselman (2013).

#### Immunohistochemistry

For immunohistochemistical staining, RBC was fixed with 4% formaldehyde in a 1:2 ratio for 20 min at room temperature and washed using 0.1 M PBS. Washed RBC were dispersed on a slide and heat fixed. Immunostaining was performed according to the detailed protocols of Grau et al. (2013) and Bizjak et al. (2015) with primary antibody dilutions of 1:150 for Rabbit anti – phospho-eNOSSerine^1177^ (Merck, Darmstadt, Germany, 07–428), 1:500 for Rabbit anti-phospho-eNOS Serine^116^ (Merck, Darmstadt, Germany, 07–357), 1:400 for Rabbit anti-phospho-eNOS Threonine^495^ (Cell Signaling, Leiden, Netherland, 9574S), 1:700 for Rabbit anti eNOS (BD Biosciences, NJ, United States, 610299), 1:500 for Rabbit anti Human AKT1/ PKBα (Merck, Darmstadt, Germany, 07–416), 1:500 for Rabbit anti-phospho AKTSerine^473^ (Cell Signaling, Leiden, Netherlands, 9271). Primary antibody was incubated for 1 h (for Serine^1177^: overnight at 4°C). A control area, located on the same slide, was incubated in the absence of primary antibodies. After rinsing with TBS and an incubation step with 3% Normal Goat Serum (Dako Agilent Technologies, Germany), both areas were incubated with the secondary goat-anti-rabbit antibody (biotinylated, dilution 1:400; Dako Agilent Technologies, Germany) for 1 h at RT. A streptavidin-horseradish-peroxidase complex (Sigma-Aldrich, United States) was applied as detection system (dilution 1:400) for 30 min at room temperature. The staining was developed using 3,3-diaminobenzidine-tetrahydrochloride solution (Sigma-Aldrich, United States) in 0.1 mol/L TBS.

The stained slides were dehydrated in raising alcohol solutions, mounted with Entellan^®^ (Merck, Darmstadt, Germany) and covered.

Pictures were taken using a Leica microscope coupled to a CCD-camera (DXC-1850P, Sony, Germany) with a magnification of 400-fold. Gray value determination was used for staining intensity analysis. The mean gray values of the edge of 50 RBC on at least 4 different visual fields of the test area and 10 RBC on at least 2 visual fields of the control area were measured with the software “Image J” (National Institutes of Health, United States).

Total immunostaining intensity was calculated as the mean of measuring RBC gray value minus mean background gray value which was obtained on three different cell free areas of the slide. Mean gray values of the control area were subtracted from mean gray values of the test area to yield net gray value representing staining of the RBC.

#### RBC S-Nitrosylation

For S-nitrosylation analysis of α- and β-spectrin, whole blood was separated by centrifugation (5000 g, 1 min, 4°C). Plasma was removed, RBC pellet was washed using 0.1 mol PBS and again centrifuged. RBC pellet was stored at -20°C until measurement.

S-nitrosylation was determined using S-Nitrosylated Protein Detection Kit (Cayman Chemicals, Ann Arbor, United States) which employs the Biotin-Switch Assay after Jaffrey and Snyder (2001). The protocol has been described in detail elsewhere (Grau et al., 2013). Using the kit buffer and solutions, RBCs were first lysed. Then, free thiol groups were blocked and S-nitrosothiols were cleaved. The newly formed thiols were biotinylated. The samples were then separated by gelelectrophoresis using 4–12% Bis-Tris gel (BioRad, Munich, Germany) and appropriate 1 × MOPS running buffer (BioRad). 60 μg of total protein were separated for 1 h under constant 90 mA and transferred to a polyvinylidene fluoride membrane (0.45 mm pore size). The background of the membrane was blocked in 2% bovine serum albumin (in 1 × TBS with 0.1% Tween 20) overnight at 4°C and incubated with a horseradish peroxidase (dilution 1:2000). The reaction was developed using an enhanced chemiluminescence kit containing peroxidase substrate (Thermo Fisher Scientific). S-nitrosylated protein bands at 240 and 220 kDa, previously identified as α-spectrin and β-spectrin, respectively (Grau et al., 2013), were examined for different “Integrated densities” using the (National Institutes of Health, Bethesda, Maryland, United States) software (Grau et al., 2013).

#### Nitrite Measurement

Whole blood was centrifuged (1000 g, 4°C, 10 min) and plasma samples were stored at -80°C for nitrite analysis.

For RBC nitrite determination, RBC were immediately mixed with preservation solution (800 mM K_3_[Fe(CN)_6_], 100 mM NEM, 10 V-% Igepal, 90 V-% aqua dest) in a 5:1 ratio and stored at -80°C until measurement (Pelletier et al., 2006). For plasma nitrite measurements, samples were thawed on ice and directly measured. For measurement of RBC nitrite, frozen samples were thawed on iced while mixed with methanol in a 1:2 ratio for protein precipitation and centrifuged at 21000 g for 10 min at 4°C. The supernatant was collected in new reaction tubes. Plasma nitrite, nitrite of the supernatant (=RBC nitrite) and nitrite levels of prepared standards were determined using an ozone-based chemiluminescence NO detector (CLD 88e, EcoPhysics, Switzerland) as described by Grau et al. (2007). Samples (100 μl) were injected into an acidified tri-iodide solution that reduces nitrite to NO gas at 60°C. The reduction solution was gas-flushed using helium as NO inert gas. The Helium-NO mix was purged in a NaOH trap and NO concentration of the samples was analyzed by the CLD system. The Chart FIA software (EcoPhysics, Switzerland) was used to analyze the area under the curve and the nitrite/NO concentrations of the samples and standards were calculated. All samples were measured in triplicate. Plasma nitrite concentrations did not require correction. Total RBC nitrite concentration of the sample was corrected for nitrite levels of methanol and preservation solution (Pelletier et al., 2006).

### Statistical Analysis

All descriptive and inferential statistical analyses were conducted using SPSS 25^®^ (IBM^®^, Armonk, NY, United States). To determine the effect of the training interventions on RBC deformability, endurance parameters, and nitrite parameters, a separate 3 × 3 (time^∗^group) mixed ANOVA with repeated measures was conducted. ANOVA assumption of homogenous variances was tested using Maulchy-test of Sphericity. Greenhouse-Geisser correction was used when a violation of Mauchly’s test was observed. Partial eta-square (η^2^_p_) values are reported as effect size estimates. If 3 × 3 mixed ANOVA revealed a significant time-point^∗^treatment or time^∗^group interaction effect on any variable, this effect was further investigated carrying out Bonferroni corrected *post hoc* pairwise comparison. Due to a lower number of samples (*n* < 10), the effect of the training interventions on RBC-NOS activation and S-Nitrosylation and the acute effect (pre, post, 24 h post) on RBC deformability was determined with the help of a student t-test or Wilcoxon test for dependent variables. Kolgomorov-smirnov test was applied to test for normal distribution.

Group differences were determined by a one-way ANOVA. Bonferroni *post hoc*-test was used to calculate significant differences between the tested groups.

For all inferential statistical analyses, significance was defined as p-value less than 0.05. Results were presented as means and standard deviations (SDs). Figures were created with Prism 6 (La Jolla, United States).

## Results

### Endurance Capacity

**FIGURE 2:**
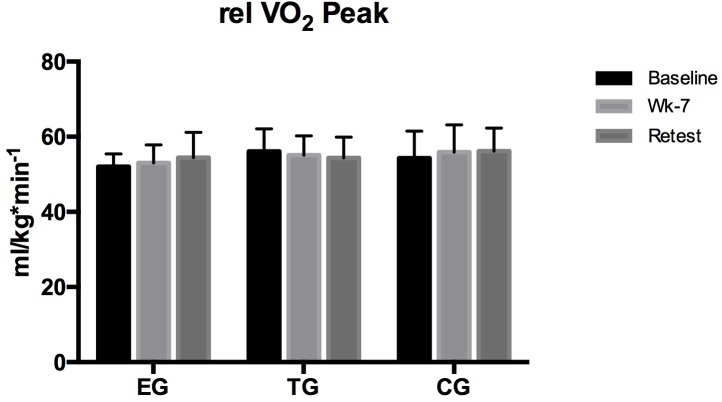
Relative maximum oxygen uptake (relVO_2_peak) at the endurance ramp-test on the treadmill in EMS-Group (EG), training-group (TG), and control-group (CG) measured before (Baseline) after week 7 (Posttest), and again after 3 weeks (Retest). Values are presented in means ± SD.

Relative VO_2_peak did not differ between the groups nor was a within group^∗^time effect observed (Figure 2).

### Basal Blood Parameters

The analysis of the training based influences on basal blood parameters revealed a significant acute increase in the number of reticulocytes from pre to 24 h post at wk-7. Group comparison showed scattered differences between the three groups. However, the differences cannot be attributed to an effect of the training interventions (Table 2).

**Table 2:** Basal blood parameters (mean ± SD).

		Baseline			Wk-7			Retest
				
		Pre	Post	24 h Post	Pre	Post	24 h Post	Pre
**Erythrocytes**	EG	5 ± 0.25	4.98 ± 0.25	5.06 ± 0.26	5.09 ± 0.23	5.03 ± 0.25	4.97 ± 0.29	4.97 ± 0.24
(10^6^/μl)	TG	5.19 ± 0.61	5.10 ± 0.59	5.19 ± 0.66	5.31 ± 0.58	4.83 ± 1.15	5.18 ± 0.66	5.16 ± 0.47
	CG	5.2 ± 0.37			5.19 ± 0.27			5.18 ± 0.35
**Thrombocytes**	EG	253.2 ± 56.7	258.8 ± 52.6	253.75 ± 55.9	258.67 ± 49.5	266.78 ± 44.2	257.67 ± 54.8	262.7 ± 46.4
(10^3^/μl)	TG	217.5 ± 42.3	219.5 ± 46.4#	223.8 ± 44.7	218.4 ± 40.4#	209.9 ± 40.9##	216.3 ± 43.5#	212.5 ± 47.2#
	CG	256.3 ± 63.5			255.44 ± 59.8			271.0 ± 63.4
**Leukocytes**	EG	5.9 ± 1.7	6.14 ± 2.46	6.39 ± 1.81	6.26 ± 1.85	5.87 ± 1.57	5.77 ± 1.49	6.23 ± 1.41
[10^3^/μl]	TG	5.64 ± 1.28	5.2 ± 0.94	5.81 ± 1.29	6.13 ± 1.28	5.46 ± 1.41	5.67 ± 1.48	5.4 ± 1.21#
	CG	7.57 ± 2.24#			6.74 ± 1.48			6.46 ± 1.62
**Reticulocytes**	EG	9.36 ± 2.94	9.91 ± 3.14	9.65 ± 3.59	8.14 ± 2.18	8.59 ± 2.31	10.01 ± 2.03*	8.39 ± 1.45
(%o)	TG	12.47 ± 4.64	11.94 ± 5.0#	12.09 ± 4.67	11.75 ± 4.13##	9.73 ± 2.67	13.11 ± 3.67	10.15 ± 5.37
	CG	10.87 ± 3.01			9.81 ± 2.55#			9.63 ± 2.04
**Hemoglobin**	EG	14.86 ± 0.67	14.78 ± 0.66	15.01 ± 0.73	15.11 ± 1.03	14.91 ± 0.90	14.61 ± 0.91	14.69 ± 0.86
(g/dl)	TG	14.56 ± 0.96	14.38 ± 1.09	14.71 ± 1.03	14.96 ± 1.13	14.29 ± 1.11	14.49 ± 1.11	14.645 ± 1.07
	CG	15.27 ± 1.05			15.05 ± 0.89			15.11 ± 1.0
**Hematocrit**	EG	43.44 ± 1.94	43.22 ± 1.98	44.13 ± 1.80	44.56 ± 2.60	43.78 ± 2.11	43.89 ± 2.42	43.11 ± 2.32
[%]	TG	42.73 ± 2.28	42.18 ± 2.52	43.18 ± 2.89	43.9 ± 3.07	42.4 ± 2.59	43.4 ± 2.99	42.91 ± 2.63
	CG	45.00 ± 2.96			44.75 ± 1.49		44.57 ± 2.64	44.44 ± 2.74
**MCV**	EG	87.11 ± 3.69	86.89 ± 4.04	87.00 ± 3.51	87.44 ± 3.68	87.33 ± 3.57	87.89 ± 3.44	87.22 ± 3.7
(fl)	TG	83.74 ± 8.21	82.82 ± 7.65	83.81 ± 8.16	83.8 ± 8.64	83.1 ± 8.5	84.4 ± 8.49	83.55 ± 8.17
	CG	86.56 ± 2.74			86.78 ± 2.33			86.22 ± 2.22
**MCH**	EG	29.89 ± 1.69	29.78 ± 1.56	29.43 ± 1.81	29.67 ± 1.66	29.67 ± 1.66	29.56 ± 1.59	29.67 ± 1.66
(pg)	TG	28.45 ± 3.08	28.36 ± 3.07	28.73 ± 3.10	28.4 ± 2.24	28.1 ± 3.11	28.3 ± 3.23	28.64 ± 3.17
	CG	29.33 ± 0.87			29.22 ± 0.83			29.33 ± 0.87
**MCHC**	EG	34.22 ± 0.83	34.33 ± 1.00	34.29 ± 1.25	34.0 ± 0.87	34.33 ± 0.71	33.67 ± 0.87	34.0 ± 0.71
(g/dl)	TG	33.91 ± 0.83	34.27 ± 0.9	34.09 ± 0.83	33.8 ± 0.79	33.4 ± 1.65	33.0 ± 1.25	34.18 ± 1.17
	CG	33.78 ± 0.67			33.78 ± 1.09			34.0 ± 0.71


*^∗^*P* < 0.05 vs. Pre; *#P* < 0.05 vs. EG; *##P* < 0.01 vs. EG.*

### RBC Proportion

**FIGURE 3:**
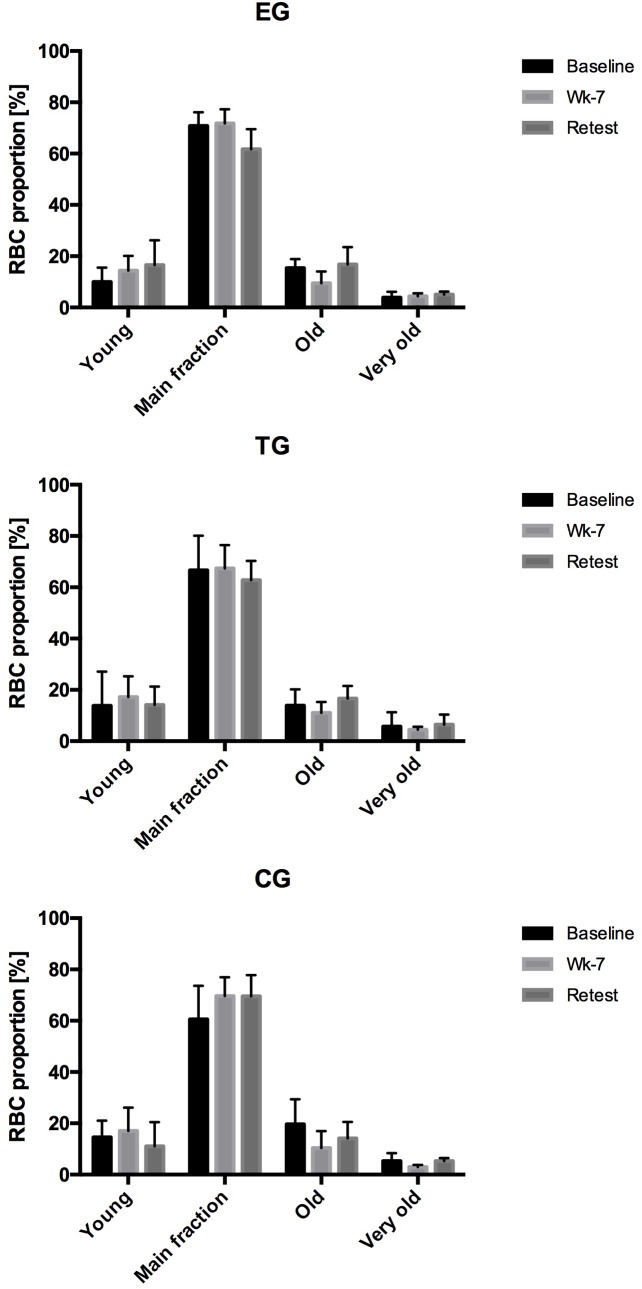
Changes (%) in the RBC proportion of the different RBC subfractions (Young, Main fraction, Old, and Very old) at rest (Pre-values) in EG, TG, and CG measured at Baseline in week 7 (Wk-7) and again after 3 weeks (Retest). Values are presented in means ± SD.

Analysis of the proportion of young RBC, main fraction, old RBC and very old RBC of EG, TG and CG during the study period revealed a slight increase in young RBC in EG from Baseline to Retest. In parallel, proportion of main fraction decreased from Baseline to Retest in EG. No changes were observed for the old and very old RBC fraction of this study group. In TG and CG, RBC proportion remained unaltered during the study period (Figure 3).

### RBC Deformability

#### Total RBC

**FIGURE 4:**
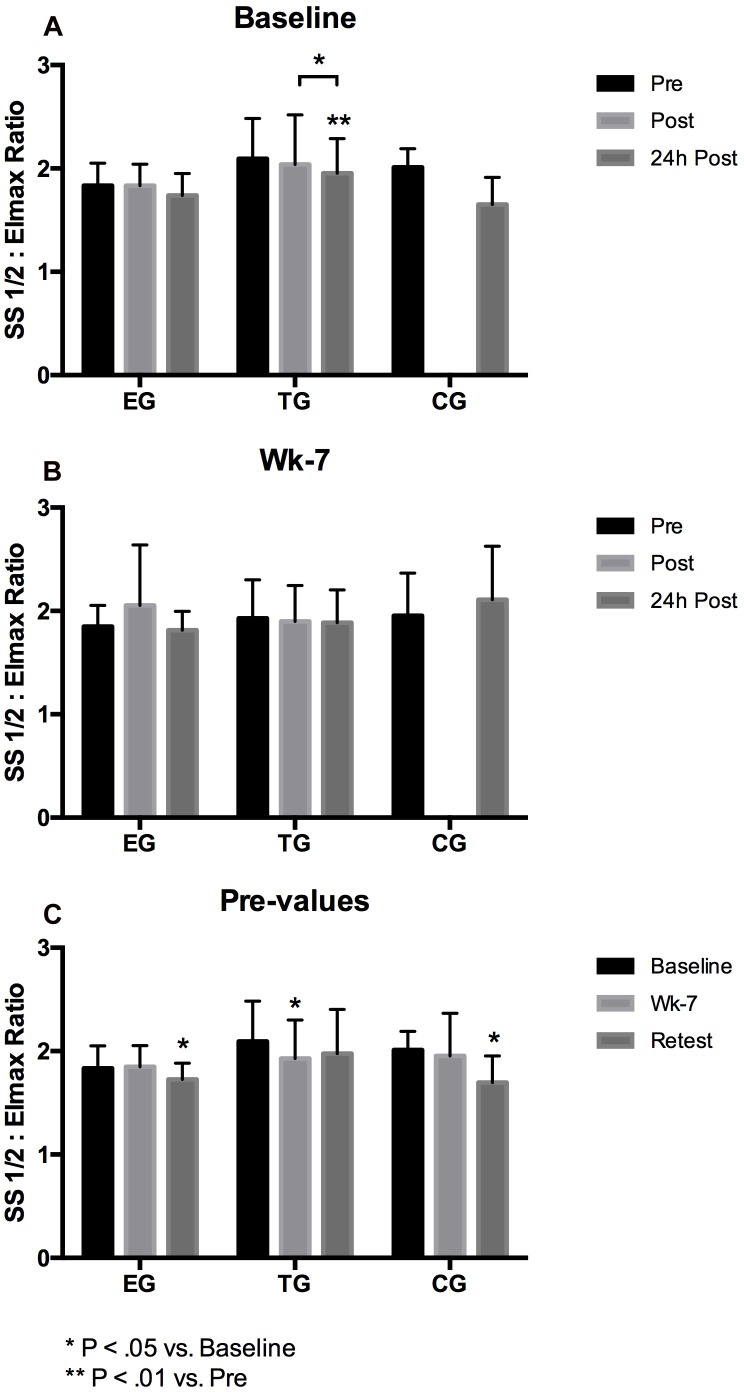
Acute changes in RBC deformability presented as SS1/2: Eimax Ratio in EG, TG, and CG measured before (Pre) 15–30 min after (Post), and 24 h after (24 h Post) the intervention at **(A)** Baseline and **(B)** in week 7 (Wk-7). Chronic changes of the Pre values over time at **(C)** Baseline, Wk-7 and after 3 weeks without intervention (Retest). Values are presented in means ± SD.

The analysis of the acute effects of WB-EMS on RBC deformability revealed a significant decrease in deformability Ratio for TG from pre to 24 h post (*p* = 0.005) and from post to 24 h post (*p* = 0.028). No significant changes were shown at wk-7 within the groups (Figure 4).

The 3 × 3 mixed ANOVA on total RBC pre-values revealed a significant main effect over time (F = 8.420, d = 2, *p* = 0.001, η^2^_p_ = 0.260) but no time^∗^group effect. Significant interaction effect on total RBC deformability Ratio was further analyzed by *post hoc* comparisons revealing a significant decrease of the Ratio from Baseline to Retest for EG (*p* = 0.019) and for CG (*p* = 0.017), and for TG from Baseline to wk-7 (*p* = 0.033).

Group comparison showed no differences between the three groups within the study period.

#### Young RBC

**FIGURE 5:**
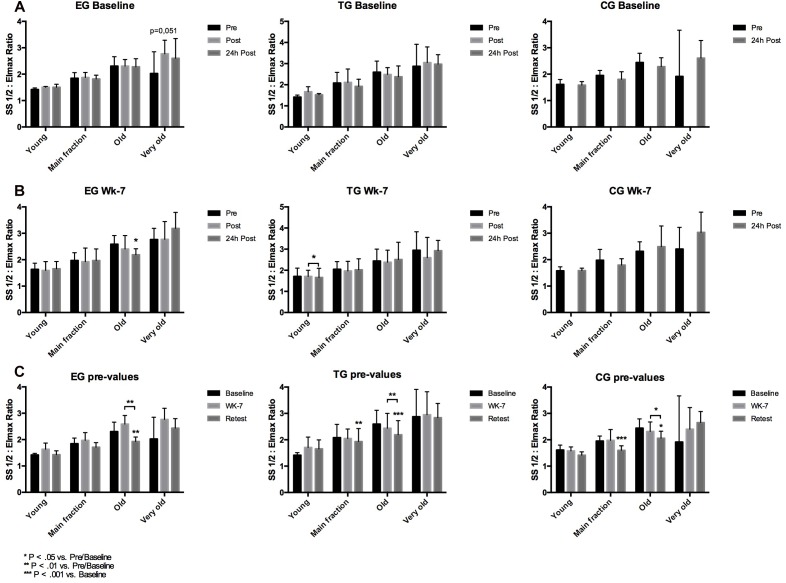
Acute changes in RBC deformability presented as SS1/2: Eimax Ratio in on the different subfractions (Young, Main fraction, Old, and Very old) in (horizontal) EG, TG, and CG measured before (Pre) 15–30 min after (Post), and 24 h after (24 h Post) the intervention at **(A)** Baseline and **(B)** in week 7 (Wk-7). Chronic changes of the Pre values of the different subfractions over time **(C)** at Baseline, Wk-7, and after 3 weeks without intervention (Retest). Values are presented in means ± SD.

At Baseline, statistical analysis revealed no acute changes between pre, post, and 24 h post in the RBC deformability Ratio of young RBC and no difference between the groups. At wk-7, Ratio significantly decreased from post to 24 h post in TG (*p* = 0.028). Data remained unaltered in EG and TG, respectively (Figure 5). Pre value comparisons revealed no significant time or time^∗^group effects.

#### Main Fraction

At Baseline and wk-7, Ratio showed no significant differences between the three tested groups. Analysis of pre-values revealed a significant main effect over time for the three groups (*F* = 15.807; *d* = 1,324, *p* < 0.001, η^2^_p_ = 0.397) but no group^∗^time effect. A significant chronic decrease in RBC deformability Ratio was observed in the TG (*p* = 0.005) from Baseline to Retest and for CG from Baseline to Retest (*p* > 0.001) and from wk-7 to Retest (*p* = 0.05), respectively (Figure 5).

#### Old RBC

Red blood cell deformability Ratio of old RBC showed no differences between the groups at Baseline. At wk-7, Ratio significantly decreased from pre to 24 h post in EG (*p* = 0.015). Comparison of pre values of the three time points showed a significant time effect from Baseline to Retest (*F* = 30.521, *d* = 2, *p* < 0.001, η^2^_p_ = 0.560) and a significant time^∗^group effect (*F* = 3.612, *d* = 4, *p* = 0.012, η^2^_p_ = 0.231). *Post hoc* analysis showed a significant decrease from Baseline to Retest for EG (*p* = 0.001) and TG (*p* < 0.001) and a significant decrease in all three groups from wk-7 to Retest (EG: *p* = 0.004; TG: *p* = 0.002; CG: *p* = 0.019), respectively. Values did not significantly differ between the groups at the different tests and time points (Figure 5).

#### Very Old RBC

Red blood cells deformability ratio remained unaffected during intervention, time or time^∗^group effect were not observed (Figure 5).

### RBC Protein Activation State

#### Akt Kinase

##### Total Akt kinase

**FIGURE 6:**
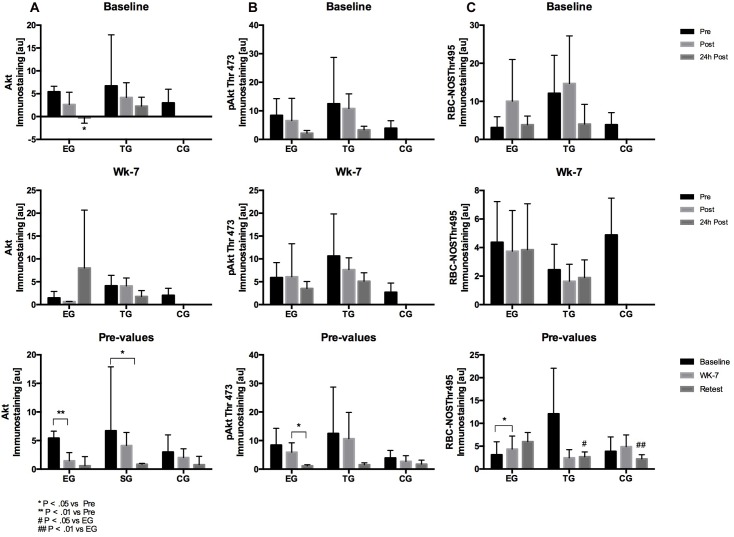
Acute activation of **(A)** Akt, **(B)** pAKtThr473, and **(C)** RBC-NOSThr495 in EG, TG, and CG before (Pre) 15–30 min after (Post), and 24 h after (24 h Post) the intervention at (vertical) Baseline and in week 7 (Wk-7) and changes of the Pre-values over time at Baseline, Wk-7, and after 3 weeks without intervention (Retest). Values are presented in means ± SD.

Statistical analysis revealed a significant decrease in total Akt at Baseline for EG from pre to 24 h post (*p* = 0.02). Values significantly decreased in EG from Baseline to wk-7 (*p* = 0.006) and for TG from Baseline to Retest (*p* = 0.022). No changes were observed for CG. Group comparison showed no differences between the groups over time (Figure 6).

##### pAkt threonine^473^

Staining intensity did not differ between pre, post, or 24 h post test of Baseline and wk-7 in each of the tested groups. The comparison of the pre values over time showed a significant decrease for EG from wk-7 to Retest (*p* = 0.02). No group differences were detected at the different tests nor within the study period (Figure 6).

#### RBC-NOS Phosphorylation Sites

##### Total RBC-NOS

**FIGURE 7:**
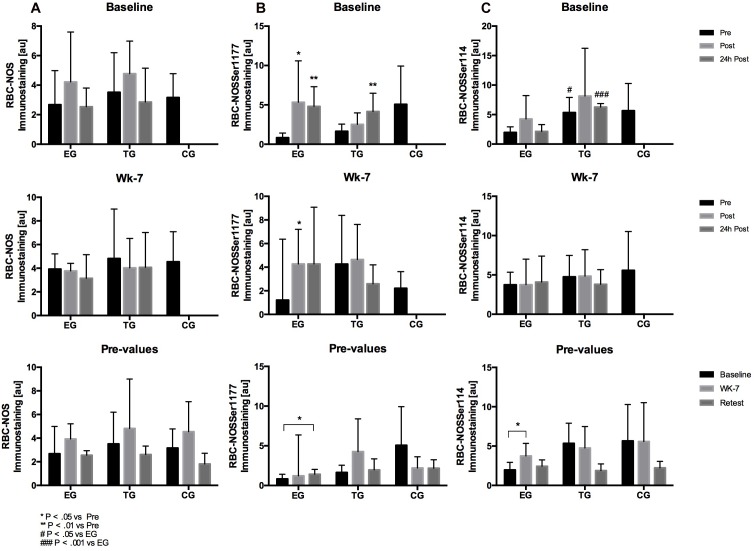
Acute activation of **(A)** RBC-NOS, **(B)** RBC-NOSSer1177, and **(C)** RBC-NOSSer114 in EG, TG, and CG before (Pre) 15–30 min after (Post), and 24 h after (24 h Post) the intervention at (vertical) Baseline and in week 7 (Wk-7) and changes of the Pre-values over time at Baseline, Wk-7 and after 3 weeks without intervention (Retest). Values are presented in means ± SD.

Total RBC-NOS showed no acute alternation in EG and TG at Baseline or wk-7, respectively. Also, total RBC-NOS signal remained unchanged during the study groups for each of the tested groups (Figure 7).

##### RBC-NOS serine^1177^

Statistical analysis revealed a significant increase in RBC-NOS serine 1177 staining intensity of EG at Baseline from pre to post (*p* = 0.04) and pre to 24 h post (*p* = 0.003) and of TG from pre to 24 h post (*p* = 0.007), respectively. At wk-7, RBC-NOS serine 1177 signal increased in EG from pre to post (*p* = 0.048). A significant increase in staining intensity was observed for EG from Baseline to Retest (*p* = 0.034). No chronic changes were shown for TG or CG (Figure 7).

##### RBC-NOS threonine^495^

Statistical analysis revealed no significant acute effects at Baseline and wk-7 for EG and TG. Comparison of pre-values over time revealed a significant increase in EG from Baseline to wk-7 (*p* = 0.014) but not for TG or CG, respectively. Group comparison revealed significant differences in the pre-values at Retest between EG and TG (*p* = 0.009), and between EG and CG (*p* = 0.004), respectively (Figure 6).

##### RBC-NOS serine^114^

Red blood cell nitric oxide synthase serine 114 signal remained unchanged at Baseline and wk-7 for EG and TG, respectively. Comparison of pre values suggest a significant increase in RBC-NOS serine 114 signal in EG from Baseline to wk-7 (*p* = 0.043). Group comparison revealed significant differences between the pre-values (*p* = 0.025) and 24 h post values (*p* = 0.0001) of EG and TG at Baseline (Figure 7).

### RBC Nitrite

**FIGURE 8:**
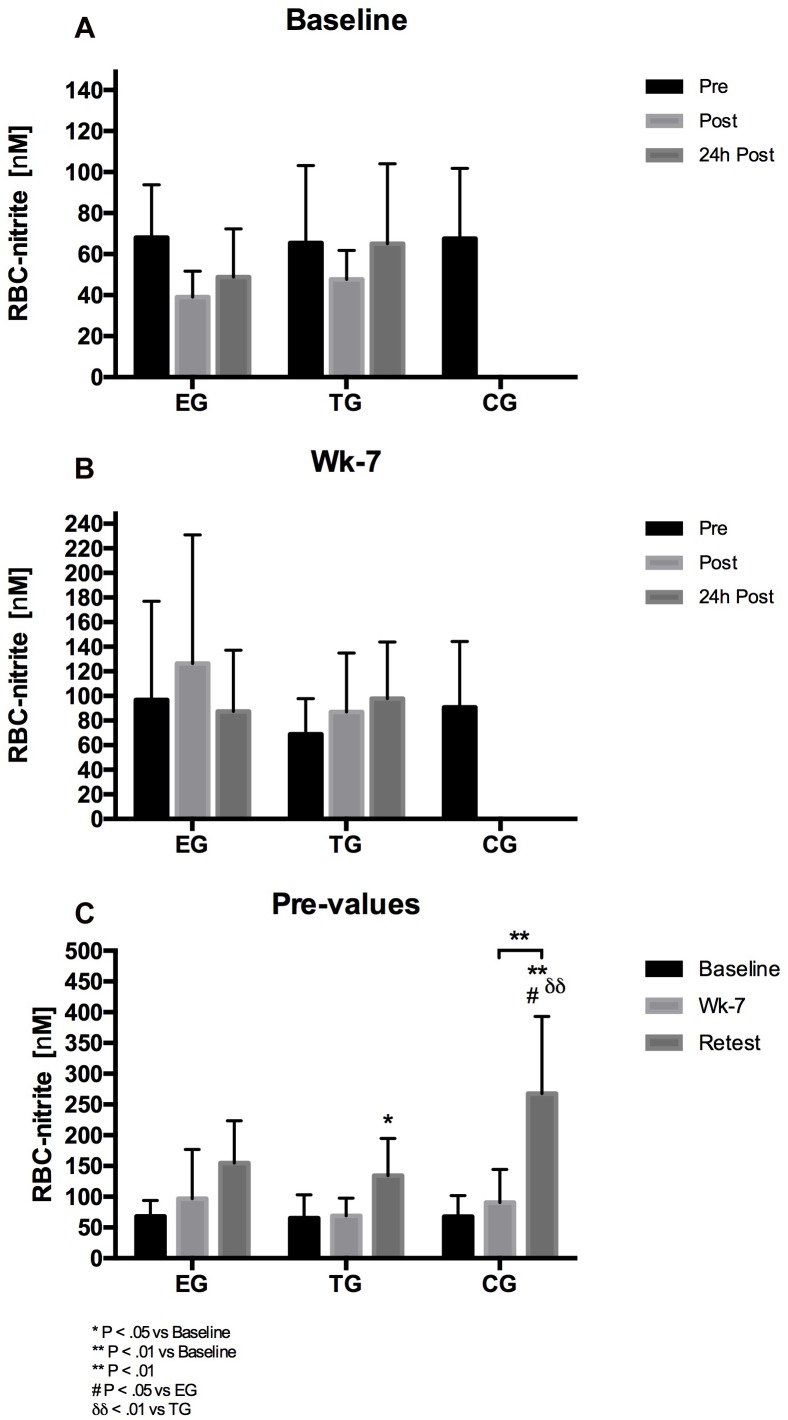
Acute changes in RBC-nitrite in EG, TG, and CG measured before (Pre) 15–30 min after (Post), and 24 h after (24 h Post) the intervention at **(A)** Baseline and **(B)** in week 7 (Wk-7). Chronic changes of the Pre values over time at **(C)** Baseline, Wk-7, and after 3 weeks without intervention (Retest). Values are presented in means ± SD.

The 3 × 3 mixed ANOVA on RBC nitrite revealed no acute effects within subjects factor time or group^∗^time effect at Baseline or wk-7, respectively. Regarding the chronic effects, the analysis of the pre-values showed a significant effect within subjects factor time (*F* = 35.728, df = 1.60, *p* < 0.001, η^2^_p_ = 0.608) and a significant group^∗^time effect (*F* = 4.373, df = 3.20, *p* = 0.009, η^2^_p_ = 0.276). The *post hoc* analysis showed a significant increase in RBC nitrite from Baseline to Retest for TG (*p* = 0.022) and CG (*p* = 0.006), respectively and from wk-7 to Retest for CG (*p* = 0.004). Group comparison revealed a significant difference at Retest between CG and EG (*p* = 0.043) and CG and TG (*p* = 0.009), respectively (Figure 8).

#### S-Nitrosylation

##### α-spectrin (240 kDa)

**FIGURE 9:**
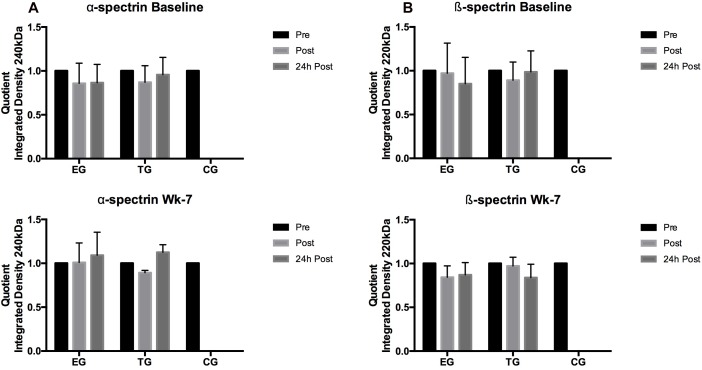
Acute changes in **(A)** α-spectrin and **(B)** β-spectrin in EG, TG, and CG measured before (Pre) 15–30 min after (Post), and 24 h after (24 h Post) the intervention at (vertical) Baseline and in week 7 (Wk-7). Values are presented in means ± SD.

Students *t*-test revealed no significant acute effects for α-spectrin at Baseline and wk-7 in EG and TG, respectively. Group comparison revealed significantly lower α-spectrin S-nitrosylation for CG at Retest compared to EG (*p* = 0.04) (Figure 9).

##### β-spectrin (220 kDa)

Similar to α-spectrin, S-nitrosylation of ß-spectrin showed no significant acute changes at Baseline and wk-7. A significant decrease in β-spectrin S-nitrosylation was observed in all three groups at Retest compared to Baseline (EG, *p* < 0.001; TG, *p* = 0.001; CG, *p* = 0.003) (Figure 9).

## Discussion

The aim of the present study was to investigate whether WB-EMS affects RBC turnover which might affect overall deformability of circulating RBC by rejuvenation of the RBC population and if this might be related to improved endurance capacity. The key findings of the investigation indicate an increase in young RBC in the EG group along with improved overall RBC deformability, represented by decreased SS1/2:EImax ratio. Detailed observation of the different RBC subfractions revealed improved RBC deformability of old RBC during study period. This improvement was not only observed in the EG but also in TG and CG. Changes in RBC deformability were not associated to altered S-nitrosylation of the spectrins. Endurance capacity remained unchanged during study period.

RBC deformability changes have been associated to influence endurance capacity (Koliamitra et al., 2017) and WB-EMS in turn has been described to affect RBC deformability. Thus, it was assumed that WB-EMS derived improvement in RBC deformability might affect endurance capacity. The results presented herein do not reflect this assumption and are thus in contrast to the findings by Amaro-Gahete et al. (2018) who describe a positive effect of WB-EMS application on runner’s VO_2_max. All tested participants were experienced soccer players and performed on a competitive level. Training volume and training intensity were not significantly altered during the intervention period and all participants showed a comparable training status to the runners (VO_2_max: 53 ml/kg^∗-1^) described by Amaro-Gahete et al. (2018). This might be explained by differences in current frequency, higher training duration and higher intensities of exercises with superimposed EMS in the cited study (Amaro-Gahete et al., 2018).

Red blood cell deformability is an important cell characteristic allowing RBC to pass the microcirculation for gas exchange. RBC deformability has been shown to be affected by a variety of factors with NO being one of them (Kleinbongard et al., 2006; Grau et al., 2013). Within RBC, NO availability depends on RBC-NOS activity (Kleinbongard et al., 2006) under normoxic conditions and nitrite reduction by deoxygenated hemoglobin under hypoxic conditions (Gladwin, 2006; Gladwin and Kim-Shapiro, 2008). RBC-NOS dependent NO generation was described to be affected by shear stress conditions, e.g., exercise, through activation of PI3-Akt kinase pathway (Suhr et al., 2012) or pharmacological stimuli such as insulin (Kleinbongard et al., 2006; Grau et al., 2013). RBC NO reaction routes include the oxidation to nitrite and nitrate, binding to hemoglobin or active cysteine thiol groups also referred to as S-nitrosylation (Ozüyaman et al., 2008). RBC-NOS generated NO was shown to increase S-nitrosylation of the cytoskeletal proteins α- and β-spectrin which was associated to increased RBC deformability (Grau et al., 2013). These reaction routes are well described for mechanical stimulation (Ulker et al., 2009, 2010; Kuck et al., 2019), endurance sports (Suhr et al., 2012; Koliamitra et al., 2017; Tomschi et al., 2018b; Bizjak et al., 2019), but also other types of sport were shown to affect RBC-NOS/NO pathway and RBC deformability (Bizjak et al., 2018). The impact of WB-EMS stimuli on the RBC-NOS/NO signaling pathway has been first shown by Filipovic et al. (2015) suggesting that WB-EMS affects RBC deformability with acute changes being explained by increased RBC-NOS activation while chronic changes in RBC deformability did not involve RBC-NOS activation. Thus, it was hypothesized for the recent study that WB-EMS affects RBC turnover and that an increase of the proportion of young RBC affects overall RBC deformability. The results of the study in part confirm the hypothesis. Proportion of young RBC increased by trend from Baseline to Retest by 65% (*p* = 0.07) while an increase of young RBC was not observed in the other tested groups. In parallel, proportion of main fraction decreased in EG by 13% (*p* < 0.05) and proportion of very old RBC increased by 30% from Baseline to Retest. Similar changes were not detected in CG and TG. The described changes were not related to possible changes of the measured blood parameters because number of measured blood cells and also RBC associated parameters, e.g., hemoglobin concentration, MCV, MCH and MCHC, remained unaltered throughout the study period. RBC deformability has been shown to be affected by RBC age with old RBC showing lowest RBC deformability values (Bizjak et al., 2015). A shift in RBC age distribution thus affects RBC deformability of overall circulating RBC pool. A recent study indicated that WB-EMS positively affects RBC deformability of professional soccer players (Filipovic et al., 2015). The recent findings indicate no acute effect of WB-EMS on RBC deformability but a significant improvement of overall circulating RBC deformability during the study period. However, an increase in overall RBC deformability was also detectable in TG and CG suggesting that the start of training and competition phase accompanied with higher training load and volume might be responsible for the changes in RBC deformability (Nader et al., 2018; Tomschi et al., 2018a). These data are thus in contrast to the findings of Filipovic et al. (2015).

Compared to the study by Filipovic et al. (2015) the soccer players in the present study had a significantly lower training volume (2–4 vs. 6–7 session/week) per week and thus had a lower fitness level. Studies have shown that moderate exercise increases deformability (Tripette et al., 2010), but intensive exercise can reduce it (Yalcin et al., 2003). As in previous studies shown EMS can be a very intense training method that can produce high metabolic and muscular stress (Jubeau et al., 2008; Nosaka et al., 2011; Filipovic et al., 2016). The applied WB-EMS stimulus might have been too intense for the players due to a lower level of fitness. Furthermore, Filipovic et al. (2015) suggested that the combination of WB-EMS stimulus and soccer specific endurance training load positively affected deformability. Accordingly, the results reveal that the training volume of only 2–4 sessions per week might have been too low to positively influence RBC deformability with two WB-EMS sessions per week.

Red Blood Cells deformability of the sub-fractions support data of the literature that RBC deformability decreases with increasing RBC age (Bizjak et al., 2015). RBC deformability of the RBC sub-fractions remained unaltered at Baseline which supports data of overall RBC suggesting that WB-EMS does not acutely affect RBC deformability. RBC deformability of the main fraction and very old RBC remained unaltered in all three study groups while RBC deformability of old RBC increased during study period. This increase was observed for all three study groups and might thus explain increased RBC deformability of overall circulating RBC. RBC-NOS activation state and RBC-NOS dependent NO production were shown to affect RBC deformability through S-nitrosylation of cytoskeletal spectrins (Grau et al., 2013). Thereby, RBC-NOS activation is affected by Akt kinase activation (Suhr et al., 2012). Total Akt kinase and activation of Akt kinase remained unaffected by the intervention. Total RBC-NOS content was not affected by the intervention but RBC-NOS phosphorylation at its activate residue serine 1177 increased from pre to post WB-EMS and thus support findings of Filipovic et al. (2015). Acute increases in RBC-NOS serine 1177 phosphorylation were also observed at wk-7 and comparisons of pre values suggests that WB-EMS increases RBC-NOS activation. These findings are in contrast to published data of Filipovic et al. (2015). Given the high heterogeneity of the data, it is speculated whether the documented statistical significances would have a physiological effect. Similar findings were observed for RBC-NOS phosphorylation sites serine 114 and threonine 495 which were associated to decreased RBC-NOS activation (Suhr et al., 2012; Heiss and Dirsch, 2014). Values remained unaltered during the study. RBC NO production showed no acute changes at Baseline or at wk-7. But comparisons of pre values revealed increasing values in TG and CG at wk-7 and Retest compared to Baseline, respectively. Since alterations were not found in EG, it seems unlikely that the applied WB-EMS program affects RBC-NO levels. In accordance, S-nitrosylation of the spectrins also remained unaffected.

Limitations of the present study include the lower training volume compared to our previous investigation with professional soccer players (Filipovic et al., 2015). This might have a major influence on deformability because RBC deformability is affected by exercise with increasing training load resulting in increased deformability. Thus, the results of the two studies are difficult to compare. Regarding training load in soccer, the differences in playing time, high intensity running, and/or sprint distances during soccer match and training might show high deviations between the players that might affect adaptive processes. Further, the players were only advised to maintain usual food intake but nutrition was not controlled. An unbalance diet of some players also could have influenced adaptations or performance.

In summary, the effect of WB-EMS on RBC physiology seems to be rather low and results are only in part comparable to previous findings (Filipovic et al., 2015). Because performance parameters also remained unaltered in the recent study, it can be speculated that the combination of WB-EMS and soccer specific training load were lower compared to previous studies and thus too low to induce changes in RBC physiology.

## Data Availability

The datasets for this manuscript are not publicly available because of legal reasons. Requests to access the datasets should be directed to the corresponding author.

## Ethics Statement

This study was carried out in accordance with the recommendations of the Ethics Committee of the German Sports University Cologne. All subjects gave written informed consent in accordance with the Declaration of Helsinki. The protocol was approved by the Ethics Committee of the German Sports University Cologne [06-02-2014].

## Author Contributions

AF, MG, and WB conceived and designed the research. AF conducted the experiments. DB and FT prepared, processed, and measured the parameters. AF and MG analyzed the data and wrote the manuscript. DB, FT, and WB revised the manuscript. All authors read and approved the manuscript.

## Conflict of Interest Statement

The authors declare that the research was conducted in the absence of any commercial or financial relationships that could be construed as a potential conflict of interest.
